# 

**Published:** 1884-02

**Authors:** 


### CONTENTS.

COMMUNICATIONS.

Pyorrhea Alveolaris—The Result of Disease.................................. 59
Report on the Teeth of a Mound-Builder.................................. 82
On the Treatment of Pulpless Teeth.................................. 88

SELECTIONS.

Reflex Nervous Influence.................................. 95
Replantation and Transplantation of Teeth.................................. 98
Temperance and Longevity.................................. 99
Old Shoes.................................. 101
Antiquity of the Trephine.................................. 101
The Immediate Suture of Divided Nerves.................................. 101
Salmagundi.................................. 102

EDITORIAL.

Mississippi Valley Dental Society.................................. 106
To be Continued.................................. 108
Commencement.................................. 108
College Associations.................................. 109
Book Notices.................................. 109
Scientific American.................................. 110

